# Editorial: Biobehavioral and social pathways linking childhood adversity and health across the lifespan

**DOI:** 10.3389/fpsyg.2022.992562

**Published:** 2022-08-23

**Authors:** Susanne R. de Rooij, Annie T. Ginty, Katherine B. Ehrlich, Neha A. John-Henderson

**Affiliations:** ^1^Epidemiology and Data Science, Amsterdam UMC, University of Amsterdam, Amsterdam, Netherlands; ^2^Amsterdam Public Health Research Institute, Aging & Later Life, Health Behaviors & Chronic Diseases, Amsterdam, Netherlands; ^3^Amsterdam Reproduction and Development, Amsterdam, Netherlands; ^4^Department of Psychology and Neuroscience, Baylor University, Waco, TX, United States; ^5^Center for Family Research, University of Georgia, Athens, GA, United States; ^6^Department of Psychology, University of Georgia, Athens, GA, United States; ^7^Department of Psychology, Montana State University, Bozeman, MT, United States

**Keywords:** childhood adversity, biobehavioural and social pathways, biobehavioural, protective factors, health risk

Adverse events in childhood are linked to mental and physical health well into old age. Beginning with the landmark study by Felitti et al. (1998), and with dozens of subsequent follow-up investigations in the decades since, we have ample evidence for a strong and graded association between adverse childhood experiences (ACEs) and multiple risk factors for several of the leading causes of death in adults. This pattern is especially worrisome, as we know that ACEs are common, affecting an estimated two thirds of the US population, with a potentially higher prevalence in middle- and low-income countries (Merrick et al., 2018; Kidman et al., 2020). The COVID-19 pandemic has exacerbated these trends, with evidence that children's exposure to adversity has increased as a consequence of the pandemic (Calvano et al., 2021).

Despite abundant evidence for the negative health effects of ACEs, less is known about the biobehavioral and social pathways through which ACEs impart these enduring negative effects. The purpose of this Research Topic was to collect articles that shed light on these potential pathways. With a better understanding of these pathways, it will be possible to identify promising points of focus for interventions seeking to reduce the negative consequences of childhood adversity for later health.

This Research Topic includes nine articles which collectively paint a picture of the complex and multifaceted relationship between ACEs and psychological, behavioral, social, and biological variables. For example, Novais et al. showed that ACEs affect a wide range of health-related problems. Relative to individuals who had not experienced adversity in childhood, individuals with adversity exposures in childhood had higher total scores for more risk behaviors and health conditions, ranging from adverse mental health as well as adverse cardiometabolic outcomes. Similarly, Willemen et al. showed that a higher number of child maltreatment types, as well as distinct types of maltreatment such as emotional neglect, emotional abuse and sexual abuse were associated with a greater risk for current depressed mood. In this study, C-reactive protein (CRP), an index of low-grade inflammation, was evaluated as potential mediator between child maltreatment and comorbid depression and metabolic syndrome. However, contrary to expectations, CRP was not related to child maltreatment.

Chen et al. reviewed the evidence for immune and epigenetic pathways as promising candidates that may underlie the link between childhood adversity and subsequent adverse health outcomes. They conclude that childhood adversity, ranging from child abuse and neglect to poor parent-child relationships, to low socioeconomic status, can negatively shape immune and epigenetic pathways across the lifespan, with widespread implications for mental and physical health. Counts et al. studied acute cortisol reactivity to stress as a potential pathway linking childhood adversity and health. They found that blunted cortisol was related to childhood adversity, in part due to greater threat appraisals and lower challenge appraisals. Traces of having experienced childhood threat can also been found in resting state functioning of the brain as demonstrated by Banihashemi et al. who showed that greater childhood threat was associated with lower functional connectivity between several areas in the brain controlling stress responses. Evidence from a study by Iffland and Neuner suggests that it is likely that attentional biases in the aftermath of victimization put individuals at risk for the development of psychopathology. The work by Kogan et al. shows that, for young adults from disadvantaged backgrounds, high levels of planful self-control promote positive psychosocial outcomes but simultaneously confer vulnerabilities to chronic metabolic diseases. A final pathway from ACEs to adverse health outcomes may be through sleep. ACEs have been demonstrated to be associated with adult sleep disorders (Kajeepeta et al., 2015) and in this Research Topic Yang et al. show that sleep quality is associated with life satisfaction, and this association is mediated by perceived stress and depressive symptoms.

Sleep then may be a potential pathway to intervene on in trying to mitigate adverse health effects of ACEs. Another potential target for intervention has been studied by Bleil et al. who suggest that targeting parenting quality may reduce the negative effects of ACEs on health. They describe findings from a number of family-based intervention studies, which have provided promising evidence for the possibility that improving parenting quality for children who experience adversity may yield benefits for children's cardiometabolic health.

Collectively, the findings described in this Research Topic highlight a number of different pathways that help explain the negative health effects of ACEs as well as potential targets for behavioral and psychosocial interventions. While the work in this collection focuses primarily on pathways which may contribute to the poor health outcomes linked to childhood adversity, future work should elucidate sources of resilience for individuals who experienced adversity in childhood. Understanding protective factors is essential to inform intervention efforts aiming to offset the health risks associated with childhood adversity. Because children's exposure to adversity may shape health via many divergent pathways, successful interventions may require a multipronged approach that targets several vulnerabilities and enhances protective factors simultaneously. We are encouraged by the findings of Bleil et al. which suggests that interventions might be able to mitigate some of the sequelae associated with early life adversity.

## Author contributions

All authors listed have made a substantial, direct, and intellectual contribution to the work and approved it for publication.

## Conflict of interest

The authors declare that the research was conducted in the absence of any commercial or financial relationships that could be construed as a potential conflict of interest.

## Publisher's note

All claims expressed in this article are solely those of the authors and do not necessarily represent those of their affiliated organizations, or those of the publisher, the editors and the reviewers. Any product that may be evaluated in this article, or claim that may be made by its manufacturer, is not guaranteed or endorsed by the publisher.

## References

[B1] Calvano C. Engelke L. Di Bella J. Kindermann J. Renneberg B. Winter S. M. (2021). Families in the COVID-19 pandemic: parental stress, parent mental health and the occurrence of adverse childhood experiences-results of a representative survey in Germany. Eur Child Adolesc Psychiatry. 31:1–13. 10.1007/s00787-021-01739-0 33646416 PMC7917379

[B2] Felitti V. J. Anda R. F. Nordenberg D. Williamson D. F. Spitz A. M. Edwards V.. (1998). Relationship of childhood abuse and household dysfunction to many of the leading causes of death in adults. The adverse childhood experiences (ACE) study. Am J Prev Med. 14:245–58. 10.1016/S0749-3797(98)00017-8 9635069

[B3] Kajeepeta S. Gelaye B. Jackson C. L. Williams M. A. (2015). Adverse childhood experiences are associated with adult sleep disorders: a systematic review. Sleep Med. 16:320–330. 10.1016/j.sleep.2014.12.013 25777485 PMC4635027

[B4] Kidman R. Piccolo L. R. Kohler H. P. (2020). Adverse childhood experiences: prevalence and association with adolescent health in Malawi. Am. J. Preventive Med. 58, 285–293. 10.1016/j.amepre.2019.08.028 31810632 PMC6981018

[B5] Merrick M. T. Ford D. C. Ports K. A. Guinn A. S. (2018). Prevalence of adverse childhood experiences from the 2011-2014 behavioral risk factor surveillance system in 23 states. JAMA Pediatr. 172:1038–44. 10.1001/jamapediatrics.2018.2537 30242348 PMC6248156

